# Antimicrobial Drug-resistant *Salmonella* Typhimurium (Reply to Helms)

**DOI:** 10.3201/eid0910.020716

**Published:** 2003-10

**Authors:** Jan Dahl

**Affiliations:** * Danish Bacon and Meat Council, Copenhagen, Denmark

**In Reply to Helms:** In the article by Helms et al., Helms concludes that infections with *Salmonella* Typhimurium strains resistant to ampicillin, chloramphenicol, streptomycin, sulfonamide, and tetracycline (hereafter referred to as penta-resistant) were associated with higher death rates than infections with non–penta-resistant *S.* Typhimurium. Helms also concluded that infections with quinolone-resistant (nalidixine-resistant) *S.* Typhimurium were associated with higher death rates than quinolone-susceptible *S.* Typhimurium (*1*).

Table 2 in Helms’ article provides information that enables close scrutiny of this conclusion and comparison of the excess mortality associated with penta-resistant, quinolone-susceptible *S.* Typhimurium with the excess mortality of non–penta-resistant *S.* Typhimurium (*1*). In this letter, the Table is based on the original table. However, two additional comparisons have been added: the p values, which are not based on the data but are approximations based on the parameters in the table.

**Table 2:** Two-year relative death rate of patients infected with *Salmonella* Typhimurium, by antimicrobial susceptibility pattern. Registry linkage study including 2,047 patients and a random matched sample of 20,456 people from the Danish general population

	Resistant		Susceptible^a^		
	Deaths/cases		RR^b^ (95% CI^c^)		
	31/1,094	2.4 (1.6-3.7)	Deaths/cases	RR (95% CI)	p-value^d^
Resistant to >1 drug	13/393	3.5 (1.7-7.2)	28/953	2.3 (1.5-3.5)	0.86
Ampicillin	16/347	5.1 (2.6-10.2)	46/1,654	2.1 (1.5-3.0)	0.21
Chloramphenicol	13/458	2.1 (1.1-4.1)	43/1,700	1.9 (1.4-2.8)	0.01
Streptomycin	31/969	2.5 (1.6-3.9)	46/1,589	2.4 (1.7-3.4)	0.76
Sulfonamides	15/513	2.2 (1.2-4.1)	28/1,078	2.1 (1.4-3.3)	0.60
Tetracycline	3/108		44/1,534	2.4 (1.7-3.4)	0.85
Kanamycin	5/83	10.3(2.8-37.8)	23/1,018	3.9 (2.2-6.7)	
Quinolone	12/283	4.8 (2.2-10.5)	54/1,964	2.1 (1.6-3.0)	0.02
R-type ACSSuT	5/40	13.1 (3.3-51.9)	47/1,764	2.1 (1.5-2.9)	0.06
R-type ACSSuTNx	5/40	13.1 (3.3-51.9)	7/243^e^	2.9^­^ (1.1-7.9)	0.09




^a^Susceptible refers to strains susceptible to the given antimicrobial drugs or combination of antimicrobial drugs; first cell refers to pansusceptible strains. ^b^Death rate relative to the general population, as estimated by conditional proportional hazards regression analysis, controlling for underlying illness; RR=rate ratio. ^c^CI=confidence interval. ^d^p-value for test of homogeneity, i.e., RR for resistant being the same as for susceptible strains. ^e^Strains with R-type ACSSuT, but quinolone sensitve.

The conclusion is that only quinolone resistance is associated with excess mortality compared with nonresistant isolates. Penta-resistant, quinolone-susceptible *S.* Typhimurium has a risk ratio of 2.9 (1.1 to 7.9) compared to the ratio of non–penta-resistant isolates 2.1 (1.5 to 2.9). When these figures are compared, the approximate p value is 0.55, which, of course, is far from being significant. Thus, on the basis of the article by Helms, penta resistance may not pose a greater threat to human health than non–penta resistance. However, the measured effect of penta resistance is achieved by the inclusion of quinolone-resistant *S.* Typhimurium in the group.

**Table:** Table showing additional comparisons (1)^a^

	Resistant		Susceptible		p value	
	Deaths/cases	RR^b^ (95% CI)	Deaths/cases	RR^b^ (95% CI)		
Penta with and without quinolone	12/283	4.8 (2.2 to 10.5)	47/1,764	2.1 (1.5 to 2.9)	0,06	
Penta with quinolone	5/40	13.1 (3.3 to 51.9)	47/1,764	2.1 (1.5 to 2.9)^c^	0,01^d^	
Penta without quinolone	7/243	2.9 (1.1 to 7.9)	47/1764	2.1 (1.5 to 2.9)^c^	0,55c^d^	

^a^RR, relative risk; CI, confidence interval. ^b^Adjusted for coexisting conditions. ^c^Compared to the non-penta group. ^d^Approximations based on the parameters from the table.

## References

[1] Helms M, Vastrup P, Gerner-Smidt P, Mølbak K. Excess mortality associated with antimicrobial drug-resistant *Salmonella* Typhimurium. Emerg Infect Dis. 2002;8:490–5. 11996684 10.3201/eid0805.010267 PMC2732497

